# NADPH Oxidase Biology and the Regulation of Tyrosine Kinase Receptor Signaling and Cancer Drug Cytotoxicity

**DOI:** 10.3390/ijms14023683

**Published:** 2013-02-07

**Authors:** Rafael Paletta-Silva, Nathália Rocco-Machado, José Roberto Meyer-Fernandes

**Affiliations:** 1 Clinical Research Coordination, Nacional Institute of Cancer (INCA), André Cavalcanti Street, 37, Rio de Janeiro, RJ 20231-050, Brazil; 2 Institute of Medical Biochemistry, Federal University of Rio de Janeiro (UFRJ), CCS, Bloco H, University City, Fundão Island, Rio de Janeiro, RJ 21941-590, Brazil; 3 Institute of National Science and Technology of Structural Biology and Bioimage (INCTBEB), CCS, Bloco H, University City, Fundão Island, Rio de Janeiro, RJ 21941-590, Brazil

**Keywords:** NADPH oxidase, reactive oxygen species, cancer, tyrosine kinase receptors, cancer drugs

## Abstract

The outdated idea that reactive oxygen species (ROS) are only dangerous products of cellular metabolism, causing toxic and mutagenic effects on cellular components, is being replaced by the view that ROS have several important functions in cell signaling. In aerobic organisms, ROS can be generated from different sources, including the mitochondrial electron transport chain, xanthine oxidase, myeloperoxidase, and lipoxygenase, but the only enzyme family that produces ROS as its main product is the NADPH oxidase family (NOX enzymes). These transfer electrons from NADPH (converting it to NADP^−^) to oxygen to make O_2_^•−^. Due to their stability, the products of NADPH oxidase, hydrogen peroxide, and superoxide are considered the most favorable ROS to act as signaling molecules. Transcription factors that regulate gene expression involved in carcinogenesis are modulated by NADPH oxidase, and it has emerged as a promising target for cancer therapies. The present review discusses the mechanisms by which NADPH oxidase regulates signal transduction pathways in view of tyrosine kinase receptors, which are pivotal to regulating the hallmarks of cancer, and how ROS mediate the cytotoxicity of several cancer drugs employed in clinical practice.

## 1. Introduction

Reactive oxygen species (ROS) are formed during aerobic metabolism and are much more reactive than molecular oxygen. For many years, they were only associated with cell injury, but currently, they are viewed as important components of the biological processes [1–5]. In aerobic organisms, ROS can be generated from different sources, including the mitochondrial electron transport chain, xanthine oxidase, myeloperoxidase, lipoxygenase, and uncoupled endothelial NO synthase, but the only enzyme family that produces ROS as its main product is the nicotinamide adenine dinucleotide phosphate (NADPH) oxidase family (NOX enzymes) [6]. The NOX family comprises seven members: NOX 1–5 and the dual oxidases (DUOXs) DUOX 1 and DUOX 2. The NADPH oxidase complexes were initially thought to be specific to phagocyte cells, but these enzymes are now recognized as being almost universally expressed in nonphagocytic cells.

Currently, NADPH oxidase-derived ROS are known to modulate a variety of functions in different cell types. There is increasing interest in the investigation of redox-sensitive signaling pathways, as cancer cells present high oxidative metabolism with high ROS content. Such signals involve transcription factors, such as AP-1 and NF-kappaB, and cell signaling components, such as p38 MAPKs, PKC and PI3K/AKT, which are all NADPH-derived ROS sensitive. Our goal in this review is to discuss the mechanisms by which NADPH oxidase regulates signal transduction pathways in view of tyrosine kinase receptors, a pivotal regulator of hallmarks of cancer, and the role of NADPH oxidase in the mechanisms of action of cancer drugs.

## 2. The NOX Family

**Figure 1:**
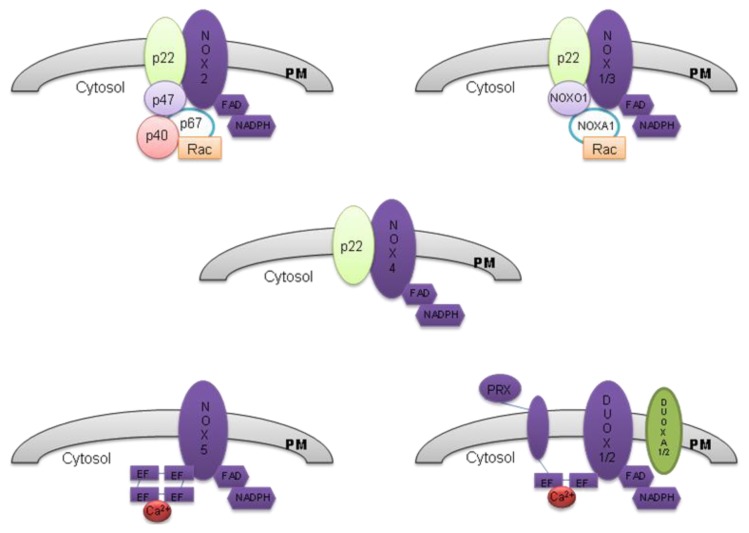
Schematic sequence of NOX isoforms and their regulatory subunits.

The NOX family consists of seven members: NOX 1–5 and two dual oxidases (DUOXs), DUOX 1 and DUOX 2. All NOX isoforms have six transmembrane domains in the *N*-terminal half, four conserved histidine residues located in the third and fifth transmembrane helices, which coordinate two hemes, and a flavoprotein domain and NADPH-binding domain in the *C*-terminal cytosolic region. NOX 5 also has Ca^2+^-binding domains, EF-hands, at the *N*-terminus. In addition to EF-hands, DUOX enzymes have a membrane-spanning region and a peroxidase-like domain at the *N*-terminus [6,7]. Figure 1 and Table 1 show the seven isoforms of NADPH oxidases, their subunits and regulators.

Different NOX proteins are often expressed in the same cell or tissue. Although they produce the same type of ROS, the enzymes regulate distinct functions in different cell types. These findings suggest that the complement of NOX proteins within a cell and their subcellular localization and coupling to external stimuli are determinants of the response to NOX activation.

### 2.1. NOX 2

The classical NADPH oxidase in phagocytes was the first member of the family to be described [26]. It consists of two membrane-bound elements, gp91phox (“phox” stands for phagocyte oxidase) and p22phox; three cytosolic proteins, p40phox, p47phox, p67phox; and a small G-protein, Rac [10].

Gp91phox is the catalytic subunit, also called NOX 2, and has a *C*-terminal cytosolic region that contains a flavoprotein domain, which is homologous to flavoprotein dehydrogenase flavin adenine dinucleotide (FAD)-binding sequences and a sequence that represents a NADPH-binding site [8,27]. Protein kinase C (PKC) has been shown to be involved in the phosphorylation of gp91phox during the activation of human neutrophils [11]. This subunit forms a heterodimer with p22phox at the plasma membrane, which is called flavocytochrome b558 [6–8].

P22phox has a proline-rich sequence (PRR) that is involved in binding to Src homology 3 (SH3) domains in the cytosolic subunit p47phox. In the resting state, the SH3 domains of p47phox bind the autoinhibitory region (AIR) in the *C*-terminal half, interrupting binding to p22phox. In the presence of an appropriate stimulus, such as phorbol 12-myristate 13-acetate (PMA), serine residues of p47phox can be phosphorylated through PKC [12], and p47phox becomes available to bind p22phox. It was also shown that tumor necrosis factor (TNF)-α induces p47phox phosphorylation in human neutrophils [13]. The p22phox subunit can be phosphorylated by PKC and phosphatidic acid on threonine residues [7,8,14].

Another cytosolic component, p67phox, is associated with the *C*-terminal PRR of p47phox through its *C*-terminal SH3 domain. It also has four tetratricopeptide-rich regions that are responsible for binding Rac proteins and a proline-rich region. The *C*-terminal domain contains the phox and Bem1p (PB1) domain, whose function is to create heterodimers between proteins containing the PB1 domain. P67phox is phosphorylated on its serine and threonine residues by PKC-dependent and -independent pathways [15].

P40phox, which is also localized in the cytosol, is associated with p67phox via mutual phox and PB1 domains. It can be phosphorylated at the serine 315 and threonine 154 residues by PKC [16], and it is not necessary to activate the oxidase complex.

The translocation of cytosolic components and their association with the membrane subunit lead to the activation of the enzyme [7,8]. NOX 2 is essential in innate host defense by both producing ROS to attack invaders and acting as a signaling molecule to initiate inflammatory and immunoprotective responses [28]. It is expressed not only in phagocytes but also vascular cells [29], the endothelium, fibroblasts, cardiomyocytes, skeletal muscle, hepatocytes, and hematopoietic stem cells [30]. NOX 2 activity dysregulation may lead to dysfunction and contribute to hypertension [17,31].

### 2.2. NOX 1

NOX 1 was the first homologue of gp91phox to be described, and it was originally isolated from colon epithelial cells (CECs) [32,33] but is also found at lower levels in vascular smooth cells, endothelial cells, the uterus, placenta, prostate, osteoclasts, retinal pericytes and macrophages [8,9,29]. There are two proposed roles for the physiological action of NOX1 in CECs: immune defense and cell proliferation. From a pathological view, the misregulation of the enzyme could be responsible for inflammatory bowel disease and carcinogenesis [17,34,35].

This isoform is composed of the membrane subunit p22phox; two cytosolic subunits, the 67phox homologue NOXA1 (NOX activator 1) and the p47phox homologue NOXO1 (NOX organizer 1); and Rac1. Its expression is induced by vasoactive factors, such as angiotensin II (Ang II) and platelet-derived growth factor (PDGF) [8,9].

### 2.3. NOX 3

NOX 3 was discovered in 2000 [36], and it is expressed in the inner ear, lung endothelial cells and fetal spleen, kidney, lung, and skull, suggesting that NOX3 plays an important role in tissue development but is turned off in adult tissue. The activity of this subunit depends on p22phox, NOXO1, NOXA1 and Rac1 [8,17–19].

### 2.4. NOX 4

NOX 4 was first identified in the kidney [37,38] but was subsequently found in many cell types, such as smooth cells, endothelial cells, fibroblasts, keratinocytes, osteoclasts, neurons, and hepatocytes. NOX 4 is constitutively and highly expressed in various cell lineages [17,29].

This isoform requires only the p22phox subunit to exert its enzymatic activity [20]; however, unlike NOX 1, NOX 2 and NOX 3, it does not require the organizer subunit-interacting proline-rich domain of p22phox [39]. Recently a p22phox-interacting protein, polymerase delta-interacting protein (Poldip2), was described to enhance the activity of NOX 4 [21]. The enzyme has been suggested to be involved in stress signals in the kidney and smooth muscle, TGFβ-induced differentiation, insulin signalling, oxygen sensing, cardiac differentiation and transcriptional regulation [17].

### 2.5. NOX 5

NOX 5 is a calcium (Ca^2+^)-dependent homologue, and it has four Ca^2+^-binding domains (EF-hands) in the *N*-terminus. This isoform has been identified in lymphoid tissues, testes, the spleen, and endothelial cells. Unlike other NOX proteins, NOX 5 does not need any other subunit for its activation; however, the enzyme is regulated by intracellular Ca^2+^ levels [7,8]. It was reported that phosphatidylinositol (4,5)-bisphosphate (Ptdlns(4,5)p_2_) plays a key role in the translocation of NOX 5 to the plasma membrane [22]. NOX 5 can also be activated by PKC through the phosphorylation of serine residues. This activation process has no equivalent in the NOX family and seems to occur on regions of this enzyme that are different from other isoforms of NADPH oxidase [40].

This enzyme participates in endothelial cell proliferation, migration and angiogenesis [8]. NOX 5 has been observed in certain pathologies, such as hairy cell leukemia [41], melanoma cells [42], prostate cancer cells [43], and Barrett’s mucosa [44].

### 2.6. DUOX 1 and DUOX 2

The dual oxidases DUOX 1 and DUOX 2 were first described as homologues of NOX 2 in the thyroid gland [45,46]. They are composed of a NOX-like region in the *C*-terminal half, two EF-hands, a membrane-spanning region and a prolonged peroxidase-like *N*-terminus. The name dual oxidase is based on the presence of an extracellular peroxidase-like domain in addition to their NOX-like NADPH oxidase core; however, no clear peroxidase activity has been attributed to human DUOXs [23,47].

DUOXs do not require other components of NADPH oxidase for their activity, but they are Ca^2+^ dependent and also need the DUOX maturation factors DUOXA1 and DUOXA2. DUOXAs are transmembrane glycoproteins characterized as endoplasmic reticulum (ER) proteins that are essential for ER-to-Golgi transition, maturation, and targeting DUOX 1 and DUOX 2 to the plasma membrane as functional complexes [7,24]. DUOX 1 is expressed in the thyroid gland, airway epithelia, placenta, prostate, testis, pancreas and heart. DUOX 2 is expressed in the thyroid gland, airway epithelia, epithelial cells in salivary excretory ducts and rectal glands [7].

In the airway epithelia, DUOX 1 gene expression is induced by Th2 cytokines (IL-4 and IL-13), and DUOX 2 gene expression is induced by Th1 cytokines (IFN-γ) [48]. Differential DUOX regulation might be important for host defense and inflammatory responses [7]. Mutations in the DUOX 2 gene in the thyroid have reported in congenital hypothyroidism [49]. At the post-translational level, both enzymes are regulated by different intracellular signaling cascades. DUOX 1 responds to cAMP and PKA-mediated phosphorylation, and DUOX2 is stimulated by the PLC cascade and PKC-dependent phosphorylation [25].

## 3. Reactive Oxygen Species

ROS are produced naturally or through metabolic dysfunction. They are responsible for phagocytosis and the regulation of cellular growth, signaling and the synthesis of important substances. However, in excess, they can be detrimental.

Superoxide (O_2_^•−^), the main ROS produced *in vivo*, is highly reactive and short lived. Therefore, it has to be produced in proximity to its target to be effective as a signaling molecule. O_2_^•−^ is capable of reacting with nitric oxide (NO), forming peroxynitrite (OONO^−^), a strong oxidizing, nitrating and nitrosylating agent. O_2_^•−^ also reacts with (FeS)_4_ clusters within proteins, which may release ferric irons, and protein thiols, such as cysteine residues. O_2_^•−^ is not able to cross biological membranes due to its negative charge and can be dismutated to hydrogen peroxide (H_2_O_2_) spontaneously or enzymatically via superoxide dismutase (SOD) [17,50,51]. H_2_O_2_ is able to cross biological membranes, is more stable than O_2_^•−^ and is regulated by catalase and glutathione (GSH) peroxidase, which converts H_2_O_2_ to water and other metabolites. The reaction of H_2_O_2_ with chloride (Cl^−^), catalyzed by myeloperoxidase, produces the oxidant hypochlorite (ClO^−^). Reaction with metal ions, such as copper (Cu^+^) and iron (Fe^2+^), generates hydroxyl radical (·HO), which reacts rapidly and indiscriminately with biomolecules of all classes. H_2_O_2_ can also reversibly react with cysteine residues on proteins, leading to their activation or inactivation, directly activate redox-sensitive kinases, such as protein kinase B, protein kinase C, the mitogen-activated protein kinase family and Janus kinase, and have a direct effect on ion channels or receptors [17,51,52].

### 3.1. NOX-Derived ROS

As mentioned earlier, NADPH oxidases produce ROS as their main product and not as a consequence, as in the case of other sources, such as the mitochondrial electron transport chain, xanthine oxidase, myeloperoxidase, lipoxygenase and uncoupled endothelial NO synthase, which produce ROS as a side effect of their action. The existence of many NOX isoforms suggests the relevance of redox-sensitive signaling cascades [51,53,54]. NADPH oxidases produce ROS by transferring an electron from NADPH to oxygen via FAD and the two heme groups, with the second heme group being responsible for reducing molecular oxygen. Heme is an obligate electron donor; therefore, it is generally accepted that O_2_^•−^ is the initial product of NADPH oxidases, although it was previously reported that NOX4 and DUOXs directly produce hydrogen peroxide (H_2_O_2_) [24,55,56].

Studies on NOX4 ROS production are controversial. In Serrander *et al.* [40], NOX4 induced a strong signal with probes that detected extracellular H_2_O_2_ but not with probes that detected extracellular O_2_^•−^. These authors also used the DHE method, which is widely used to detect intracellular O_2_^•−^, and no NOX4 signal was detected. Another method to measure intracellular O_2_^•−^, the reduction of NBT to formazan, was also used. Induced NOX4 cells elicited significant NBT reduction compared with non-induced cells. As only O_2_^•−^ is capable of reducing NBT, this result provides strong support for NOX4 O_2_^•−^ production. The study suggested that NOX4 produces primarily O_2_^•−^ in a tight intracellular compartment devoid of DNA, and this O_2_^•−^ is rapidly converted into H_2_O_2_. Others believe that NOX4 produces primarily H_2_O_2_. Dikalov *et al.* [57] showed that siRNA against NOX4 does not reduce O_2_^•−^ but does reduce H_2_O_2_ production, and Takac (2011) [56] identified the external E-loop of the protein as an essential structure for H_2_O_2_ production and showed that alterations of the E-loop switch in NOX4 led to O_2_^•−^ production.

DOUX1 and DUOX2 were originally found to produce mainly H_2_O_2_ [58]. No homology was found in the DUOXs with the E-loop of NOX4, but they have a long NH_2_ extracellular domain that might be involved in H_2_O_2_ production [24]. The DUOX maturation factors have also been associated with the type of ROS produced. When DUOX1 and DUOX2 are coexpressed with DUOX, they are not retained as ER resident proteins. The DUOX-DUOXA complex migrates to the plasma membrane and produces high amounts of H_2_O_2_ but no detectable superoxide [55]. Alterations of DUOX maturation factors switch the enzyme from H_2_O_2_ to O_2_^•−^ formation [24,56].

## 4. Regulation of Cancer Cell Biology by NADPH Oxidase Activity: Implications in Hallmarks of Cancer

The role of ROS-dependent NADPH oxidase in biological systems can be classified into at least two functions: (1) promoting oxidative stress; an imbalance between the generation and neutralization of ROS in cells has deleterious effects on macromolecules, such as proteins, nucleic acids and lipids, leading to cellular damage and enhancing the risk of mutations; and (2) the regulation of several signaling pathways that are redox sensitive contributes to cancer pathophysiology [59,60].

**Figure 2:**
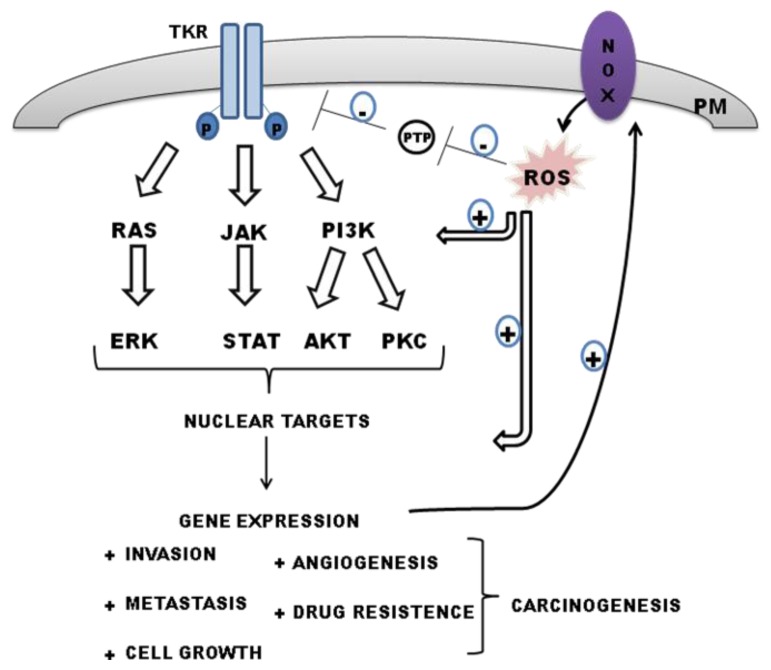
Reactive oxygen species (ROS) can act in a synergic manner with tyrosine kinase receptor (TKR) to promote carcinogenesis. TKR can enhance the expression/activity of NOX isoforms. The ROS generated from NOX activity can sustain the activation of TKR signaling pathways and of transcription factors involved on carcinogenesis through inhibition of PTP activities.

ROS-derived NADPH oxidase activity is closely related to several mechanisms underlying cancer cell biology and, consequently, disease progression. The high metabolic oxidative stress observed in cancer cells modulates a wide range of processes that confer acquired capabilities during tumor development. It has been demonstrated that all the hallmarks of cancer that were classified by Hanahan and Weinberg [61] and reviewed [62] (e.g., sustaining proliferative signaling, evading growth suppressors, resisting cell death, enabling replicative immortality, inducing angiogenesis, activating invasion and metastasis, reprogramming energy metabolism, and evading immune responses) can be modulated by ROS derived from NADPH oxidase isoform activities in a redox-regulated manner. NOX dictates carcinogenesis through the regulation of several cell signaling pathways related to carcinogenesis that respond to stress signals, such as Janus kinase-signal transducer and activator of transcription (JAK-STAT), protein kinase C, mitogen-activated protein kinase (MAPK), AKT [60,63] (Figure 2).

The effects of NOX on the hallmarks of cancer mentioned above have been well documented in recent reports [59,64]. In the following sections, we will present and discuss the role of NOX enzymes in cancer disease in view of tyrosine kinase receptor (TKR) signaling, which has been implicated in pivotal hallmarks of cancer and is currently being explored as cancer molecular target therapy.

## 5. Redox-Sensitive Regulation of Signaling Pathways: NADPH-Derived ROS as Mediators

Thiol redox regulation dictates gene expression, interfering with transcription factor activities, and can also act as a second messenger, interfering with protein functions responsible for transducing cell signaling events. Thiol group modifications are thought to play a central role in the cellular signaling process through ROS modifications. In general, the mechanisms underlying such regulation are dictated by redox reactions, which alter the cysteine residue state in protein phosphatases and kinases [65].

TKRs are one of the most important redox-responsive signaling cascades that are commonly enhanced in several cancers cells compared with normal cells. Enhanced signal transduction caused by growth factors, such as epidermal growth factor (EGF) and platelet-derived growth factor (PDGF), cytokines and hormones results in an uncontrolled proliferative response. These events are involved in the enhancement of the signaling response through an overall increase in tyrosine phosphorylation and inhibition of protein tyrosine phosphatases (PTPs) by oxidation [66]. In this context, it has been accepted that ROS derived from NADPH oxidase activity is closely related to enhancing signal transduction engaged by the activation of TKRs or inhibiting tyrosine phosphatase activity, which is responsible for inactivating TKRs or TKR-associated proteins.

### 5.1. NOX Modulates PTP Activities: Maintaining TKR Signaling by ROS

PTPs contain an essential cysteine residue that is responsible for interacting with phosphate as mechanisms of catalysis (dephosphorylating). This residue is rapidly oxidized by hydrogen peroxide, resulting in reversible enzyme inactivation. As cancer cells have high oxidative metabolism, in most cases, PTPs are in an inactive state, contributing to the maintenance of TKR activation and consequently promoting carcinogenesis events.

NOX regulates the activity of PTP1B, PTEN, SHP1/2 and low-molecular-weight protein tyrosine phosphatase (LMW-PTP) [59] and sustains TKR phosphorylation and the activation of EGFR downstream proteins. PTEN is involved in regulating a variety of cellular functions, including the cell cycle, apoptosis, DNA repair, signal transduction and cell adhesion [67]. PTEN inactivation by hydrogen peroxide occurs by oxidizing Cys-124 in the catalytic site followed by the formation of a disulphide with Cys-71 [68]. Hydrogen peroxide-derived NOX1 inactivates PTEN activity. As PTEN dephosphorylates PIP3 to PIP2, PIP3 accumulation sustains cell signal transduction mediated by PIP3/AKT [69]. In prostate cancer, hydrogen peroxide enhances migration and invasion by inactivating PTEN and increasing the expression of the chemoattractant receptor CXCR-4 [70]. PTPs are also involved in anti-apoptotic effects and prosurvival events; in pancreatic cancer cells, low-molecular-weight phosphatases are inhibited by NOX4, leading to sustained activation of the JAK2 pathway [71]. Leukemic B cells (hairy cell cancer) express NOX5 and produce ROS in a calcium-dependent manner that does not involve PKC. Furthermore, NOX5 colocalization with SHP-1 inhibits phosphatase activity, which contributes to maintaining cells in active states by sustaining phosphorylation of the CD22 receptor [41].

## 6. Interplay between Tyrosine Kinase Receptor Signalling and ROS-Derived NADPH in Regulating Carcinogenesis

### 6.1. Epidermal Growth Factor Receptor (EGFR)

EGFR is a member of the ErbB family of tyrosine kinase receptors and is commonly up-regulated in non-small cell lung cancer (NSCLC), head and neck cancer, colorectal cancer and hepatocellular cancer. In general, EGFR mutation is correlated with a poor clinical prognosis. The molecular analysis of EGFR gene mutations is used as a guideline protocol to determine the prescription of EGF signaling tyrosine kinase inhibitors, such as erlotinib, and monoclonal antibodies (mAbs) against EGFR, such as cetuximab (chimeric mAb) and panitumumab (humanised mAb), as single agents or associated with standard chemotherapy and/or radiotherapy protocols [72,73].

The enhanced EGFR signaling observed in EGFR-mutated cancer cells mediates the hallmarks of cancer in different cell types through the activation of pro-survival signaling pathways, including PI3K/Akt, MAPK and JNK [74]. Several studies have reported that NOX is upstream of those signals. NOX1 enhances the expression of EGFR signaling components and confers autocrine growth. Early hydrogen peroxide-derived NOX1 activation (15 min) is required for the phosphorylation of several EGFR signaling pathways, such as c-Src/ERKs and p38/AKT (30 min). Interestingly, the activation of EGFR components is required as a mechanism of NOX-1 regulating its own expression and the expression of an EGFR ligand (TGF-α) [75]. In leiomyoma smooth muscle cells, the inhibition of NADPH oxidase impairs MAPK signaling activation and decreases the cell proliferation response to EGF [76].

Transforming growth factor beta (TGF-β) is considered to be a liver tumor suppressor because of its ability to induce apoptosis. A link between TGF/EGFR/NOX signaling has emerged as a mechanism that dictates cell death or apoptotic cell resistance in liver cancer cells. The activation of MAPK/ERK (EGFR-related signaling) confers cell resistance to TGF-β-induced apoptosis through impairing NOX4, which is induced upstream of mitochondrial-dependent apoptotic proteins of the BCL-2 family. NOX4 knockdown impairs ROS increase and all the mitochondrial-dependent apoptotic features by regulating BIM, BMF, BCL-XL, and MCL1 levels. Inhibiting EGFR potentiates TGF-β-induced NOX4 up-regulation and increases hepatoma cell death [77–79].

The proto-oncogene RAS belongs to the G-small proteins, and the structurally related genes H-RAS, N-RAS and K-RAS codify cell signal transducers that are associated with TKRs. K-RAS is commonly overexpressed (mutated) in several tumors, especially in pancreatic cancer (95% of cases) [80]. Because patients with K-RAS mutation do not respond to EGFR inhibitors, understanding the role of RAS in cancer pathophysiology is important, and RAS are being studied as a molecular target therapy for patients who have RAS gene mutation.

A relationship between RAS and the NOX family has been demonstrated to play an important role in cancer progression. RAS can stimulate migration and proliferation in a ROS-dependent manner in colon cancer cells, and RAS signaling upregulates NOX1 expression via the MEK-ERK-GATA-6 pathway. Mutations in the GATA-6-ERK phosphorylation site abolish NOX1 upregulation and impair cell growth [81].

Rac (an activator of NOX2 and NOX4) activation represents an important downstream effecter of RAS [82]. As Rac is a potent activator of NADPH oxidase activity, some authors have suggested an interplay between Rac/NADPH oxidase and K-RAS. Inhibiting Rac1 activity (Rac1 mutant construct) inhibits the growth of mutant K-RAS human pancreatic cancer cells but not pancreatic cancer cells with wild-type K-RAS [83] in a superoxide-dependent manner. The induction of NOX activity by K-RAS was elucidated in pancreatic cancer cell lines; those that overexpressed K-RAS had significantly higher levels of superoxide than those that did not express the oncogene without diminishing antioxidant defense (superoxide dismutase). Additionally, it was demonstrated that NOX2 was only expressed in cells expressing K-RAS and that silencing NOX2 expression diminished superoxide generation and cell growth [84].

RAS/NOX also modulates cell growth and proliferation via activating the ERK/MAPK signaling pathway and cyclin D1, a regulator of the cell cycle [85].

The role of RAS and Rho (a crucial small GTPase protein that functions in the formation of stress fibers and focal adhesions) signaling on invasion/metastasis-related events was also demonstrated in view of ROS. NOX1 causes the loss of stress fiber formation and suppression of focal adhesion in a RAS-dependent manner through inhibiting Rho activity. Hydrogen peroxide-derived NOX1 inactivates LMW PTP (phosphatase), and subsequent p190RhoGAP activation causes Rho downregulation [86]. Additionally, NOX1 inhibition attenuates RAS-dependent metalloproteinase-9 expression via inhibiting IKKα kinase/NF-κB signaling [87].

The role of NOX in the activation of tyrosine receptors is even more complex due to its ability to participate in the cross talk between cell surface receptors, such as G-protein-coupled receptors (GPCRs) and RTK. GPCRs employ multiple strategies for EGFR transactivation that can be ligand dependent, which involves the release of the EGFR ligand family (TGF-α and EGF) by the action of metalloproteases (MMPs), such as TACE/ADAM, and the activation of EGFR in an autocrine/paracrine manner [88], or ligand independent, through sustaining EGFR activation due to the activation of protein kinase signaling pathways associated with the receptor. As the aim of the present review is to focus on effects of the NOX family on signaling events that participate in carcinogenesis, the complex signaling pathway of GPCRs will not be discussed. For further information, reviews addressing GPCRs and their signaling in detail have been published [88,89].

The transactivation of EGFR by NOX has been related to premalignant changes in lung cells. ROS-derived NADPH oxidase activation by tobacco smoke induces metalloproteinase TACE activation in a Src/PKCɛ-dependent manner, which induces the processing and release of amphiregulin and EGFR activation [90]. In colon rectal cancer, c-Src induces the expression of NOX1 in a Rac1-dependent manner [91]. In NSCLC, EGFR transactivation is also mediated by ROS-dependent NADPH oxidase activity/c-Src, which results in the downstream activation of the PI3K/AKT/NF-κB pathway and cyclooxygenase-2, which are molecular predictors of the overall survival of patients with NSCLC [92].

The human formyl peptide receptor (FPR) and FPR-like 1 (FPRL1) belong to the GPCR family and mediate the transactivation of EGFR through NOX-dependent ROS generation. In a human lung adenocarcinoma cell line (CaLu-6), FPRL1 activated NADPH oxidase in an ERK-dependent manner. Superoxide derived from the NADPH oxidase activation of c-Src (TYR-416), a known EGFR activator [93], transactivates EGFR/JAK/STAT3, promoting cell proliferation [94].

The bombesin receptor family (BBR) is another GPCR involved in regulating carcinogenesis. BB2R and BB3R stimulate ERK and EGFR phosphorylation by stimulating metalloproteases and the release of TGF-α in a c-Src-dependent manner in head and neck cancer cell (HNSCC) lines and NSCLC in an NADPH oxidase-dependent manner [95].

### 6.2. Vascular Endothelial Growth Factor Receptor (VEGFR)

The major role of VEGFR is to induce angiogenesis and support glucose uptake in cancer cells. The induction of new vessel formation by VEGF plays a critical role in supplying the high metabolic demand of cancer cells. Similar to EGFR, disrupting VEGFR signaling pathways has been commonly used as molecular target therapy for highly vascularised tumors, such as colorectal and hepatic cancer [96,97], lung cancer [98], and breast cancer [99].

The induction of VEGF gene expression by hypoxia in tumor cells involves both an increase in the rate of gene transcription, which is mediated by the transcription factor hypoxia-inducible factor (HIF), and an enhancement of the expression and stability of VEGF and/or VEGFR mRNA [100,101]. In this context, NADPH oxidase isoforms present an important feature in regulating new blood vessel formation downstream of VEGFR signaling and mediating HIF-1 expression in a ROS-dependent manner. Human microvascular endothelial cells overexpressing Nox4 present an increase in VEGF and HIF-1 in an insulin-dependent AKT phosphorylation manner. Downregulating NOX4 impairs endothelial cell migration and proliferation and, consequently, angiogenesis [102].

In cancer, NOX4-dependent ROS production and HIF-1 activation are observed in the hypoxic region of glioblastoma tumors [103]. The VEGFR/NOX4-dependent accumulation of HIF-1 surrounding melanoma tumors is correlated with chemotherapy and radiotherapy resistance [104]. In prostate cancer cell models, PKCδ activation increases HIF and VEGF levels in an NADPH oxidase dependent-manner, implying a role for ROS-derived NADPH oxidase [105]. The stability of HIF-1 expression is mediated by p22^phox^-dependent mTOR activation in renal cancer cell models [106].

In K-RAS transformed rat kidney cells, NOX1 mediates angiogenesis, enhancing VEGF expression through the phosphorylation and activation of a transcription factor, SP-1, which binds to the VEGF promoter [107]. In leukemic cells, Rac-1 and AKT activate NOX2 and NOX4 in response to VEGF, and ROS-derived NOX activities sustain VEGFR-2 receptor phosphorylation and support glucose uptake [108]. In endothelioma cells, VEGF stimuli upregulate NOX1, but not NOX2 or NOX4. NOX1 expression mediates angiogenic effects through activating NF-κB and inhibiting PPARα [109], which inhibits pro-angiogenic effects by blocking VEGFR-2 expression [110].

## 7. NOX and Cancer Drug Therapy: Implications of ROS-Mediated Cytotoxicity and Resistance

Several studies have linked ROS-derived NADPH oxidase activity with chemotherapy- or cancer drug-induced cytotoxicity in clinical practice or under investigation (pre-clinical and clinical trial studies). The mechanism underlying NADPH oxidase-mediated cancer drug toxicity or resistance remains poorly understood, but interestingly, the results of several studies strongly suggest an important role of ROS derived from NADPH oxidase activity.

### 7.1. ROS-Derived NADPH Oxidase Mediates Drug Cancer Cytotoxicity

Several chemotherapeutics stimulate NOX-dependent ROS production as a mechanism to mediate cell killing [111]. Platinum-based chemotherapy is extensively used in several guideline protocols for several clinical cancer manifestations. Platinum-derived drugs inhibit or impair thioredoxin reductase enzyme activity, which leads to enhanced oxidative stress inside cells [112]. Paclitaxel, a microtubule-targeting agent, increases Rac1 expression and NOX activity. An interesting result of a previous study showed that superoxide generated in paclitaxel-treated cells was directly released outside the cells, where it was subsequently dismutated into hydrogen peroxide, and that extracellular hydrogen peroxide could exert cytotoxic bystander effects (mediate cytotoxicity to cells that were not directly exposed to paclitaxel) [113].

Monoclonal antibodies and tyrosine kinase inhibitors have been largely used in clinical practice, and these drugs improve the survival of cancer patients in comparison with conventional chemotherapies. NOX enzymes mediate the mechanisms of action of the molecular cancer targets obinutuzumab and tositumomab (anti-CD20 mAbs used in B-cell malignancies) and their cytotoxic effects via the induction of superoxide derived from NOX2 [114]. NOX4 is upregulated in erlotinib-treated head and neck cancer cells (HNSCCs), and the increased hydrogen peroxide production is correlated with cell toxicity. HNSCCs pretreated with the anti-oxidant NAC or cells in which the NOX4 isoform is knocked down show resistance against erlotinib-induced ROS cytotoxicity [115].

Elucidating the mechanisms underlying NADPH oxidase-mediated cell death helps to understand the efficacy of cancer drug combination therapies. The association of doxorubicin (platin-based chemotherapy) and gefitinib (EGFR tyrosine kinase inhibitor) enhances hepatocarcinoma cell death through NOX4-derived superoxides through the activation of caspase-3 [116]. However, in a colorectal cancer cell model, an antagonistic effect between cetuximab and oxaliplatin was observed in view of NOX1 activity. Oxaliplatin-mediated cytotoxicity was limited by the inhibition of EGFR/NOX1-induced hydrogen peroxide production in the presence of cetuximab [117].

### 7.2. Overcaming Drug Resistance in View of ROS-Derived NADPH Oxidase

Overcoming drug resistance is a challenge in cancer therapy. Understanding the mechanisms related to cancer drug resistance therapy is needed to optimize cancer treatment and design new guideline protocols. ROS are involved in several cancer drug resistance mechanisms. P-glycoprotein (P-gp), an ABC cassette transporter, plays a major role in chemotherapeutic resistance. Overexpressing NOX1 inhibits P-gp function and overcomes the cell resistance phenotype [118]. In breast cancer, ROS mediate EGFR-tyrosine kinase inhibitor gefitinib resistance, and the hydrogen peroxide scavenger catalase was able to sensitize those cells to gefitinib treatment [119]. Interestingly, the involvement of gefitinib resistance involves signaling pathways that are redox regulated (AKT) [120]. Increased cellular antioxidants are hypothesized as a cancer drug resistance mechanism in several models. Histone deacetylase inhibitors are commonly used in T-cell lymphoma and have been reported to mediate cytotoxicity in an NADPH oxidase-dependent manner. In leukemic cells, ROS derived from NADPH oxidase increase after vorinostat and bortezomib promote cell cycle arrest, apoptosis and DNA double-stand breaks [121–123]. In contrast, increased ROS also activate the transcription factor Nfr2, which induces antioxidant expression. Combination therapy with vorinostat and a glutathione depletion agent (PEITIC) reverses the drug resistance phenotype of several leukemic cells [123]. In prostate cancer, NADPH oxidase-dependent ROS generation mediates radiotherapy resistance, and pretreating cells with NADPH oxidase inhibitors sensitizes cells to radiation [124]. However, modulating prostate cancer cell redox states with parthenolide, a sesquiterpene lactone, potentiates radiotherapy-mediated cell toxicity by diminishing anti-oxidant defense via NADPH oxidase-dependent ROS generation and activation of the PI3K/AKT/FOXO signaling pathway [125].

## 8. Conclusions

Advances in our understanding of the complex cellular and molecular mechanisms involved in regulating signaling pathways related to carcinogenesis in view of ROS have provided new insight into signaling pathway interplay and the mechanisms of action of drugs that are used in cancer treatment. In fact, several mechanisms remain poorly understood because cancer cells may present a wide range of phenotypes, which dictates different responses to cancer drugs.

Managing redox content inside cells is harmful. Due to the complex mechanisms involved in the activation of NADPH oxidases, these enzymes can be targeted at several different levels of their activity [126]. Once NADPH oxidase can regulate activities of several proteins and downstream signaling pathways, and provides the major non-mitochondrial source of ROS inside cells, it would be an important therapeutic target in cancer. To validate NADPH oxidase as a key player in the hallmarks of cancer and a molecular target for cancer, it would be interesting to determine an epidemiological profile of the expression of NADPH oxidase isoforms in cancer cells, especially those derived from samples of patients undergoing biopsy or surgery, and their correlation with cell signaling pathways and therapy used for treating the disease.

## Figures and Tables

**Table 1:** NOX isoforms, their regulatory subunits and activators.

NOX isoforms	Subunits	Regulators	References
NOX1	p22phox, NOXA1, NOXO1 and RAC1	ANG II, PDGF	[8,9]
NOX2	gp91phox, p22phox, p40phox, p47phox, p67phox, RAC1	PKC, (TNF)-α, phosphatidic acid	[7,8,10–16]
NOX3	p22phox, NOXO1, NOXA1, RAC1	Unknown	[8,17–19]
NOX4	P22phox	Poldip2	[20,21]
NOX5	NONE	Ca^2+^, ptdlns(4,5)p_2_	[7,8,22]
DUOX1	DUOXA1, DUOXA2	IL-4, IL-3,Camp, PKA	[7,23–25]
DUOX2	DUOXA1, DUOXA2	IFN-γ, PLC, PKC	[7,23–25]
